# Supplementary material for: Novel fungal metabolites as dual cholinesterase inhibitors: A computational approach for Alzheimer’s disease therapy
Source: PLoS One. 2025 Jun 16;20(6):e0326219. doi: 10.1371/journal.pone.0326219 (PMC12169564; doi:10.1371/journal.pone.0326219)
Supplement: S1 Table — (DOCX) [file pone.0326219.s001.docx]

**S1 Table.** The bioactive compounds of the fungal metabolites with their MeFSAT identifiers, PubChem ID, fungal metabolite name, chemical formula, SMILES, and chemical structures.

| MeFSAT identifier | PubChem ID | Fungal metabolite | Chemical formula | SMILES | Chemical structure |
| --- | --- | --- | --- | --- | --- |
| MSID000785 | 403923 | Fumitremorgin C | C22H25N3O3 | COc1ccc2c(c1)[nH]c1c2C[C@@H]2N([C@H]1C=C(C)C)C(=O)[C@H]1N(C2=O)CCC1 | 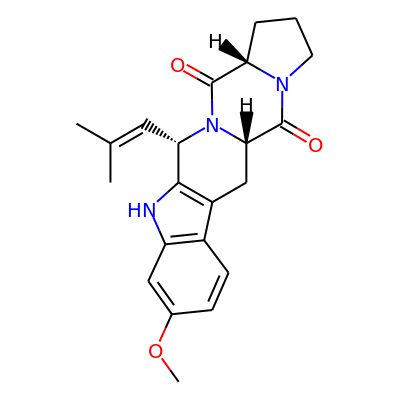 |
| MSID001018 | 44588895 | Hericenone J | C19H24O4 | COc1cc2COC(=O)c2c(c1C/C=C(/CCC=C(C)C)C)O | 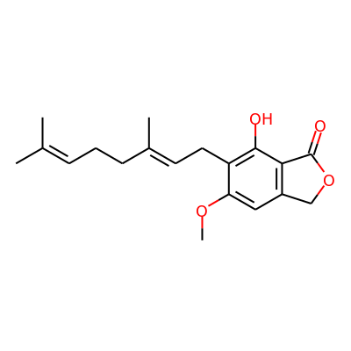 |
| MSID001208 | 76512895 | N-de(phenylethyl)isohericerin | C19H25NO3 | COc1cc2C(=O)NCc2c(c1CC=C(CCC=C(C)C)C)O | 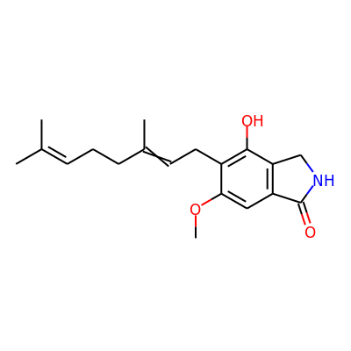 |
| MSID001127 | 53232 | Lovastatin | C24H36O5 | CC[C@@H](C(=O)O[C@H]1C[C@@H](C)C=C2[C@H]1[C@@H](CC[C@@H]1C[C@@H](O)CC(=O)O1)[C@H](C=C2)C)C | 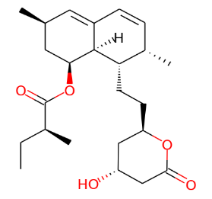 |
| MSID001011 | 11782478 | Hericenone A | C19H22O5 | COc1cc2C(=O)OCc2c(c1C/C=C(/CC(=O)C=C(C)C)C)O | 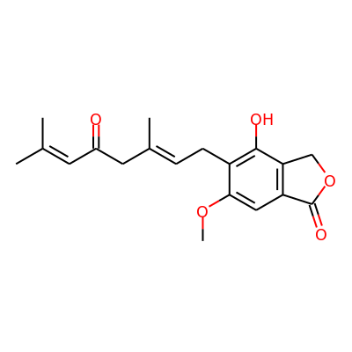 |
| MSID000666 | 132599511 | Erinacerin M | C13H15NO4 | CC([C@H]1C(=O)OCc2n1cc(C(=O)C)c(=O)c2)C | 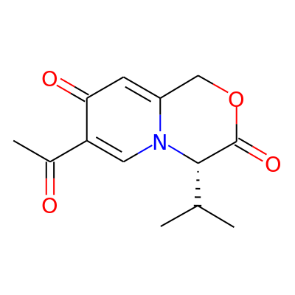 |
