# Supplementary material for: Novel fungal metabolites as dual cholinesterase inhibitors: A computational approach for Alzheimer’s disease therapy
Source: PLoS One. 2025 Jun 16;20(6):e0326219. doi: 10.1371/journal.pone.0326219 (PMC12169564; doi:10.1371/journal.pone.0326219)
Supplement: S2 Table — (DOCX) [file pone.0326219.s002.docx]

**S2 Table.** Pharmacokinetics of the fungal metabolites predicted by SwissADME.

| Properties | | Fungal metabolite | | | | | |
| --- | --- | --- | --- | --- | --- | --- | --- |
|  |  | **Fumitremorgin C** | **Hericenone J** | **Lovastatin** | **Erinacerin M** | **N-de(phenylethyl)isohericerin** | **Hericenone A** |
| Lipophilicity | Consensus Log Po/w | 2.53 | 4.23 | 3.88 | 3.33 | 4.23 | 1.12 |
| Water solubility | Log S (ESOL) | -4.18 | -4.94 | -4.57 | -3.94 | -4.22 | -2.16 |
| Pharmacokinetics | GI absorption | High | High | High | High | High | High |
|  | BBB permeant | Yes | Yes | Yes | Yes | Yes | Yes |
|  | P-gp substrate | No | No | No | No | No | No |
| Medicinal chemistry | Synthetic accessibility | 4.16 | 3.26 | 3.50 | 3.17 | 3.15 | 3.15 |
|  | Lead-likeness | Yes | Yes | Yes | Yes | Yes | Yes |
