# Supplementary material for: Novel fungal metabolites as dual cholinesterase inhibitors: A computational approach for Alzheimer’s disease therapy
Source: PLoS One. 2025 Jun 16;20(6):e0326219. doi: 10.1371/journal.pone.0326219 (PMC12169564; doi:10.1371/journal.pone.0326219)
Supplement: S3 Table — (DOCX) [file pone.0326219.s003.docx]

**S3 Table.** Drug-likeness and bioavailability of the fungal metabolites by SwissADME.

| Drug likeness properties | Fungal metabolite | | | | | |
| --- | --- | --- | --- | --- | --- | --- |
|  | **Fumitremorgin C** | **Hericenone J** | **Lovastatin** | **Erinacerin M** | **N-de(phenylethyl)isohericerin** | **Hericenone A** |
| Lipinski, Violation | Yes; 0 violation | Yes; 0 violation | Yes; 0 violation | Yes; 0 violation | Yes; 0 violation | Yes; 0 violation |
| Ghose | Yes | Yes | Yes | Yes | Yes | Yes |
| Veber | Yes | Yes | Yes | Yes | Yes | Yes |
| Egan | Yes | Yes | Yes | Yes | Yes | Yes |
| Muegge | Yes | Yes | Yes | Yes | Yes | Yes |
| Bioavailability score | 0.55 | 0.55 | 0.55 | 0.55 | 0.55 | 0.55 |
