# Supplementary material for: Novel fungal metabolites as dual cholinesterase inhibitors: A computational approach for Alzheimer’s disease therapy
Source: PLoS One. 2025 Jun 16;20(6):e0326219. doi: 10.1371/journal.pone.0326219 (PMC12169564; doi:10.1371/journal.pone.0326219)
Supplement: S4 Table — (DOCX) [file pone.0326219.s004.docx]

**S4 Table.** Toxicity analysis of the fungal metabolites by ProTox 3.0.

| Toxicity | Fungal metabolite | | | | | |
| --- | --- | --- | --- | --- | --- | --- |
|  | **Fumitremorgin C** | **Hericenone J** | **Lovastatin** | **Erinacerin M** | **N-de(phenylethyl)isohericerin** | **Hericenone A** |
| Hepatotoxicity | Inactive | Inactive | Inactive | Inactive | Inactive | Inactive |
| Carcinogenicity | Inactive | Inactive | Active | Inactive | Inactive | Inactive |
| Mutagenicity | Inactive | Inactive | Inactive | Inactive | Inactive | Inactive |
| Cytotoxicity | Inactive | Inactive | Inactive | Inactive | Inactive | Inactive |
