# Supplementary material for: Novel fungal metabolites as dual cholinesterase inhibitors: A computational approach for Alzheimer’s disease therapy
Source: PLoS One. 2025 Jun 16;20(6):e0326219. doi: 10.1371/journal.pone.0326219 (PMC12169564; doi:10.1371/journal.pone.0326219)
Supplement: Attachment — Submitted filename: Response to Reviewers.docx [file pone.0326219.s006.docx]

**ACADEMIC EDITOR:**

 The manuscript is reviewed and according to remarks after minor revision the manuscript can be accepted for publication in PLOS ONE.

- Proteins codes must be referenced form recent research articles.

**Authors’ response:** In accordance with your suggestions, we have incorporated recent research articles as references for the receptor proteins. The corresponding changes have been highlighted in yellow color.

- One of the query of reviewer is why standards were not used for docking and simulation which might help in co-relation? if possible add standards.

**Authors’ response:** We performed molecular docking studies between the receptor proteins and the control or standard drugs. However, for the molecular dynamics simulations, we used the apo structures of the receptor proteins as the standard, as it is a common practice to evaluate apo structures in comparison to their ligand-bound complexes in such studies. Studying the apo structure in molecular dynamics (MD) simulations is essential for understanding the intrinsic dynamics, flexibility, and stability of a protein in its unbound state. It serves as a baseline to compare with ligand-bound (holo) forms, helping to reveal conformational changes induced by ligand binding. Apo simulations can uncover transient or cryptic binding pockets that may not be visible in static crystal structures, which is valuable for drug discovery. Additionally, they provide insights into the structural integrity of the protein when not bound to a ligand and help elucidate potential allosteric mechanisms or conformational ensembles involved in its function.

**Reviewer #1:**

1) Was the licensed version of pymol used for the study.

**Authors’ response:** Many thanks for your contribution and critical observations on this paper. We believe, we have benefited much after going through this revision, and the manuscript’s quality has improved tremendously. Yes, we have used licensed version of pymol softaware.

2) Proteins codes must be referenced form recent research articles.

**Authors’ response:** In accordance with your suggestions, we have incorporated recent research articles as references for the receptor proteins. The corresponding changes have been highlighted in yellow color.

3) Why standards were not used for docking and simulation which might help in co-relation?

**Authors’ response:** We performed molecular docking studies between the receptor proteins and the control or standard drugs. However, for the molecular dynamics simulations, we used the apo structures of the receptor proteins as the standard, as it is a common practice to evaluate apo structures in comparison to their ligand-bound complexes in such studies. Studying the apo structure in molecular dynamics (MD) simulations is essential for understanding the intrinsic dynamics, flexibility, and stability of a protein in its unbound state. It serves as a baseline to compare with ligand-bound (holo) forms, helping to reveal conformational changes induced by ligand binding. Apo simulations can uncover transient or cryptic binding pockets that may not be visible in static crystal structures, which is valuable for drug discovery. Additionally, they provide insights into the structural integrity of the protein when not bound to a ligand and help elucidate potential allosteric mechanisms or conformational ensembles involved in its function.

The references are provided below

References:

1. Ibrahim, M.A.A., Abdeljawaad, K.A.A., Roshdy, E. *et al.* In silico drug discovery of SIRT2 inhibitors from natural source as anticancer agents. *Sci Rep* **13**, 2146 (2023). <https://doi.org/10.1038/s41598-023-28226-7>
2. Akash, S., Bayıl, I., Hossain, M.S. *et al.* Novel computational and drug design strategies for inhibition of human papillomavirus-associated cervical cancer and DNA polymerase theta receptor by Apigenin derivatives. *Sci Rep* **13**, 16565 (2023). <https://doi.org/10.1038/s41598-023-43175-x>
3. El Hassab, M.A., Eldehna, W.M., Hassan, G.S. *et al.* Multi-stage structure-based virtual screening approach combining 3D pharmacophore, docking and molecular dynamic simulation towards the identification of potential selective PARP-1 inhibitors. *BMC Chemistry* **19**, 30 (2025). <https://doi.org/10.1186/s13065-025-01389-2>

4) In-vitro results could also aid valuable findings to the study and why these were not performed?

**Authors’ response:** In-vitro experiments could undoubtedly contribute valuable mechanistic insights and serve to further substantiate the findings of this study. However, due to constraints related to resources, funds and the study’s primary focus on in-vivo or field-based outcomes, such experiments were not included in the current study. Nonetheless, we acknowledge the importance of in-vitro validation and consider it a logical next step in our future research to strengthen and expand upon the current findings.

**Reviewer #2:** The article is well written and justifying the title and objectives. The Author used all necessary tools/softwares and parameters to explore the inhibitory potential of fungal metabolites on AChE and BuChE by CADD for the better treatment of Alzheimer Disease. The pictures, tables and figures are well justified.

**Authors’ response:** Many thanks for your contribution and critical observations on this paper. We believe, we have benefited much after going through this revision, and the manuscript’s quality has improved tremendously.
